# Impact of the COVID-19 pandemic on community-based brain injury associations across Canada: a cross-sectional survey study

**DOI:** 10.3389/fpubh.2023.1166106

**Published:** 2023-11-08

**Authors:** Ana Paula Salazar, Carolina Bottari, Sophie Lecours, Michelle McDonald, Monique A. M. Gignac, Bonnie Swaine, Julia Schmidt, Carolyn Lemsky, Ashley Brosda, Lisa Engel

**Affiliations:** ^1^School of Rehabilitation, Université de Montréal, Montréal, QC, Canada; ^2^Centre for Interdisciplinary Research in Rehabilitation of Greater Montréal (CRIR), Institut universitaire sur la réadaptation en déficience physique de Montréal du CIUSSS du Centre-Sud-de-l’Île-de-Montréal, Montréal, QC, Canada; ^3^Brain Injury Canada, Ottawa, ON, Canada; ^4^Institute for Work & Health, Toronto, ON, Canada; ^5^Dalla Lana School of Public Health, University of Toronto, Toronto, ON, Canada; ^6^Department of Occupational Science and Occupational Therapy, University of British Columbia, Vancouver, BC, Canada; ^7^Community Head Injury Resource Services, Section of Brain and Therapeutics, Department of Psychiatry, University of Toronto, Toronto, ON, Canada; ^8^Brain Care Centre, Edmonton, AB, Canada; ^9^Department of Occupational Therapy, College of Rehabilitation Sciences, University of Manitoba, Winnipeg, MB, Canada

**Keywords:** COVID-19, community associations, brain injury, challenges, adaptations, public health, sustainability

## Abstract

**Background:**

The COVID-19 pandemic created new difficulties for people living with brain injury, their families, and caregivers while amplifying the challenges of community-based associations that support them. We aimed to understand the effects of the pandemic on clients who live with brain injury, as well as on the provision of community brain injury services/programs in Canada.

**Methods:**

Online cross-sectional survey conducted in January 2022. Representatives of brain injury associations across Canada completed the 31 open- and closed-ended questions about meeting clients’ needs, addressing public health guidelines, and sustaining the association. Data were analyzed using descriptive statistics (close-ended questions) and qualitative content analysis (open-ended questions).

**Results:**

Of the 45 key representatives from associations in Pacific/Western (40%), Central (56%), and Atlantic Canada (4%), the majority were paid executive directors (67%). Participants reported that the most frequent psychosocial challenges experienced by their clients during the pandemic were social isolation (98%), loneliness (96%), and anxiety (93%). To alleviate these challenges, associations implemented wellness checks and psychosocial support. Most respondents (91%) affirmed that clients faced multiple technological barriers, such as a lack of technological knowledge and financial resources for devices and/or internet. In the open-ended questions, twenty-nine (64%) associations reported providing clients with devices, technology training, and assistance. Regarding public health measures, thirty (67%) respondents reported that clients had challenges understanding and/or following public health guidelines. Forty-two associations (93%) provided tailored information to help clients understand and comply with public health measures. Although associations (67%) received pandemic-related funding from the Canadian government they still struggled with the association’s sustainability. Thirty-four (76%) lost funding or financial resources that prevented them from delivering programs or required the use of reserve funds to continue to do so. Only 56% reported receiving sufficient funding to address additional COVID-19-related expenses.

**Conclusion:**

Although the pandemic added further challenges to the sustainability of brain injury associations across Canada, they quickly adapted services/programs to respond to the increasing and varied needs of clients, while complying with protective measures. To ensure community associations’ survival it is essential to aptly recognize the vital role played by these associations within the brain injury care continuum.

## 1. Introduction

The SARS-CoV-2 virus (COVID-19) pandemic increased public health challenges for people worldwide. Physical, social, and psychological challenges were particularly distressing for individuals living with disabilities and for their caregivers or support persons (1–3). Reduced access to health, social, and community services also increased the prevalence and severity of negative outcomes in this population (4–6). Although there is a dearth of published literature specific to brain injury (i.e., stroke, traumatic or other non-traumatic brain injuries) during the current pandemic or previous public health crises, initial evidence indicates challenges and negative outcomes in community life and well-being related to the COVID-19 pandemic (5). Specifically, the pandemic rapidly worsened chronic sequelae such as physical, cognitive, mental, and behavioral, while also affecting personal finance, social support, and daily living (4, 5), which are all well documented as critical challenges for brain injury survivors (7–9). In fact, before the onset of the pandemic, the prevalence of isolation, loneliness, anxiety, depression, and other mental health issues was already higher among these individuals than in the general population (10).

Similar to other groups of people with disabilities, individuals living with brain injury were disproportionately affected by the pandemic. They were highly impacted on their health, well-being, social and community participation (4, 5) due to pre-existing health and social inequalities (1). Further, this vulnerable population faced inequities in access to public health messaging due to a lack of disability-inclusive preparedness (1, 11) with the consequences that can follow. For example, lack of clarity, inaccessible formats, and ongoing changes in public health guidelines increased stress, agitation, anxiety, and depression in people living with brain injury (4, 5) in addition to making them more susceptible to being infected (12).

Strategies to mitigate the negative consequences of public health guidelines such as offering support or services through online platforms, may not meet the needs of many brain injury survivors as digital solutions can be inaccessible or difficult to use by persons with a disability (13). Although some individuals with brain injury reported accessing health, education, and support services online with ease, others reported being unable to use needed services due to extra cognitive, technological, or economic challenges (14). Concerns exist that the digital divide increased during the pandemic, which may further impact brain injury survivors’ health, well-being, and community and social participation.

Community-based brain injury associations are essential to the community participation and well-being of thousands of people living with chronic brain injury in numerous countries. These associations play a vital role by ensuring long-term health and well-being support for brain injury survivors, their families, and caregivers, through educational, psychosocial, and daily living support programs. In Canada, these associations including national, provincial, municipal, and local societies are non-profit, that is, they receive limited or no governmental health funding and often rely on grants and fundraising activities to operate. Some evidence suggests that community-based associations serving diverse and high-need populations rapidly pivoted their services during the COVID-19 pandemic to ensure continuity of care and address newly emerging needs (15–17). These include the provision of accessible information on viral transmission prevention, online social support, mental health programming, remote daily support, and telephone helplines (16, 17). However, little is known about the impact of the COVID-19 pandemic on community-based associations from the perspective of key representatives of these associations. This information is critical to developing preparedness initiatives to optimize community brain injury association’s responses during the ongoing COVID-19 pandemic and for future public health crises. Therefore, we conducted a survey study with key representatives, of brain injury associations across Canada, to understand the effects of the COVID-19 pandemic on clients who live with brain injury, as well as on the provision of community brain injury services/programs in Canada. The specific objectives of this study were to explore the challenges experienced by people living with brain injury and brain injury associations during the pandemic and to learn the strategies or ways associations responded and adapted services to meet these challenges.

## 2. Materials and methods

### 2.1. Study design

A cross-sectional pan-Canadian online survey study was conducted in January 2022 involving community-based brain injury associations.

### 2.2. Participants

Survey respondents were key representatives (staff or volunteers) from community-based brain injury associations across Canada. Participants were included if they (1) provided signed informed consent; (2) were a staff or volunteer representative of a brain injury association; (3) had knowledge about the pre-and during-COVID-19 pandemic needs and challenges of their associations and clients, and felt they were knowledgeable about the associations’ response actions and plans to meet these needs and challenges; (4) had internet connection enabling them to participate in the survey, and (5) were able to write in English or French. Only one representative per association participated in the study (i.e., one survey per association for analysis). Representatives were not excluded for any personal characteristics such as sex, gender, or race/ethnicity. There were no exclusion criteria for which staff or volunteers could be the survey respondent.

Canadian community-based brain injury associations were identified from a list of associations^1^. Seventy associations (including small chapters) and their contact information (head of the association, e-mail, and phone number, when available) were organized by region in an Excel spreadsheet. To be included as a brain injury “association,” the organization needed to serve the brain injury population with a formal or self-reported brain injury diagnosis. Associations that were not “community-based,” that is their organization or program was part of the formal healthcare system or primarily funded by the healthcare system, were fee-for-service, and/or focused on outpatient rehabilitation, were excluded from the study. From the list of associations, two were excluded because their funding came primarily from health authorities, and one because its services were only focused on outpatient rehabilitation.

To recruit survey respondents, seven executive directors of national or provincial brain injury associations (collaborators in this project) invited key representatives from community-based brain injury associations across Canada providing services to brain injury survivors, their families, and caregivers. For larger associations, the invitation was sent to the executive directors, and for smaller associations, a paid or unpaid staff or volunteer was invited. They each received a personalized email with information about the survey, including the approximate time to complete it, the link to the informed consent, and the survey. Data were collected through the Research Electronic Data Capture System^2^ (REDCap®). Participants could contact the research team if they needed assistance.

The study was approved by the Centre for Interdisciplinary Research in Rehabilitation of Greater Montreal (2022–1424) Ethics committee and all participants provided informed consent to participate prior to completing the survey. As per the ethical approval, participants had the option to be included in a random draw that provided $500 (CAD) to their own brain injury association.

### 2.3. Survey development and content

The online survey questions were developed by an interdisciplinary team of researchers and community association co-investigators that comprised the research team (including all co-authors of this paper). The survey structure that related to the three areas of meeting the needs of clients, public health and safety, and association sustainability arose from a pilot qualitative focus group study about the first year of the pandemic (18). Thirty-one closed (multiple choice) and open-ended (free responses) questions (See Supplementary material) were designed based on the experiences of the community collaborators during the COVID-19 pandemic. The first portion of the survey consisted of 9 socio-demographic questions describing the associations (including the location of the association, number of staff, and number of clients served by the association). Next, a series of 3 questions was designed to learn about associations’ sustainability during the pandemic, key issues experienced by clients related to public health safety and access to programs, and associations’ strategies to adapt the provision of support services for people living with brain injury, their families, and caregivers (Supplementary material). For example, participants rated the extent to which they perceived challenges experienced by clients as well as to which extent their association provided services to alleviate these challenges by answering “not at all,” “to a small extent,” “somewhat,” “to a large extent,” or “to a very large extent.” They could also indicate if they did not know or did want to answer the questions (i.e., “I do not know,” “I do not want to answer”). Six open-ended questions (Question number (QN) 12, 18, 23, 24, 26, 30, and 31) invited participants to provide examples of services and adaptations provided to address the challenges. Three other open-ended questions focused on challenges faced by brain injury survivors (QN 22), advice to give to other brain injury associations (QN 29), and additional comments (QN 31).

To ensure the clarity of the questions and the usability of the survey platform, the survey was pretested and validated by a small group of collaborators who then also completed the survey as participants. Their comments led to minor modifications to clarify the wording of some questions. Before starting data collection, the survey was tested again by two team members to confirm its functionality.

The survey took approximately 30 min to complete and was available in English and French to allow participants to respond in their preferred language. Survey respondents were given a random identification code to use to report qualitative findings from open-ended questions.

### 2.4. Data analysis

Data arising from the closed-ended questions were exported into a Microsoft Excel spreadsheet and analyzed using R software (19). Descriptive statistics [i.e., frequencies and percentages, means and standard deviations (SD)] were calculated for the socio-demographic variables and all quantitative responses. For inferential statistical analyses, responses of “I do not know” or “I do not want to answer” were converted to missing values list-wise. Spearman correlations were used to calculate coefficients for variables related to the size of the associations (number of clients and number of paid staff) and meeting the needs of clients, optimizing public health safety, and sustaining brain injury associations. Correlation coefficients were interpreted as follows: 0.90 to 1.00 very high correlation, 0.70 to 0.90 high correlation, 0.50 to 0.70 moderate correlation, 0.30 to 0.50 low correlation, or 0 to 0.30 negligible correlation (20). Based on a power analysis, the sample of 60 participants was deemed sufficient at 65–90% power to detect a low-to-moderate correlation at level *α* = 0.05.

For the open-ended questions, participants’ responses were exported into Microsoft Excel and analyzed using qualitative content analysis with a deductive and inductive approach (21, 22). This content analysis was only related to the associations’ strategies to address clients’ challenges during the pandemic. We used deductive content analysis to classify the three broad categories of strategies based on a previous pilot qualitative work comprising focus groups with key representatives of brain injury associations (citation removed for blinding). Within each category, the subcategories of strategies were analyzed from an inductive approach that arose from the open-ended questions. The overall qualitative content analysis involved the following steps: (1) one researcher (AS) read the open-ended responses to become familiar with the data; (2) the same researcher coded the main ideas and (3) sorted the codes into potential categories; (4) an independent researcher (SL) validated the codes and categories; (5) a meeting with researchers and knowledge users/stakeholder co-investigators was held to present the results, and a refined version of the coding scheme was agreed upon. Disagreements in data coding were resolved through discussion between the researchers until a consensus was reached.

## 3. Results

Forty-five key representatives from 45 of the 70 brain injury associations contacted completed the online survey (response rate: 64%). Associations that responded were in Central (25; 56%), Western (18; 40%), and Atlantic (2; 4.4%) provinces of Canada (see Table 1).

**Table 1:** Characteristics of survey respondents (*n* = 45).

Variable	Frequency (%)
Region	
Northern territories (YT, NT, NU)	0 (0)
Western Canada (BC, AB, SK, MB)	18 (40%)
Central Canada (ON, QC)	25 (56%)
Atlantic Provinces (NB, NS, PE, NL)	2 (4%)
Head of the community association	
Executive director	35 (78%)
Paid staff, but not an “executive director”	5 (11%)
Volunteer or unpaid person	5 (11%)
Role in the community association	
Paid Executive director	30 (67%)
Paid staff, but not an “executive director”	7 (16%)
Volunteer or unpaid person	8 (18%)
Number of paid staff (full time and part-time)#	
Less than 5	23 (54%)
Between 5 and 10	10 (23%)
Between 11 and 20	4 (9%)
More than 20	3 (7%)
Number of clients served per year pre-pandemic†	
Less than 100	10 (23%)
Between 100 and 399	22 (49%)
More than 400	9 (20%)

YT, Yukon; NT, Northwest territories; NU, Nunavut; BC, British Columbia; AB, Alberta; SK, Saskatchewan; MB, Manitoba; ON, Ontario; QC, Quebec; NB, New Brunswick; NS, Nova Scotia; PE, Price Edward; NL, Newfoundland & Labrador. #One association had no paid staff, and four participants responded “I do not know” or “I do not want to answer.” †Four participants responded “I do not know” or “I do not want to answer”.

Most of the associations were led by a paid executive director (35; 78%) and provided direct services to people living with brain injury (44; 98%), their caregivers (35; 78%), their families (36; 80%), and/or others (12; 27%). Thirty (67%) participants were paid executive directors with the majority (35; 78%) being involved with community associations for more than 5 years.

When asked if participants thought the gender identity of their clients influenced their experiences during the COVID-19 pandemic (Q23-Supplementary material), most of the participants responded that they did not know or did not want to answer. Only seven (16%) responded “yes.” Regarding new programs tailored to specific groups of clients during the pandemic, twenty-five (56%) associations reported having services for caregivers of people living with brain injury, twenty-one (47%) for people living with mild and moderate to severe brain injury. Seventeen associations (38%) had specific programs for older clients, while thirteen (29%) offered tailored support for younger clients. Nine participants (20%) reported that their association offered specific programs for clients who identify as women, seven (16%) had programs for clients who identify as men, while only three (7%) reported having services tailored for the LGBTQI2SA+ community.

**Figure 1:**
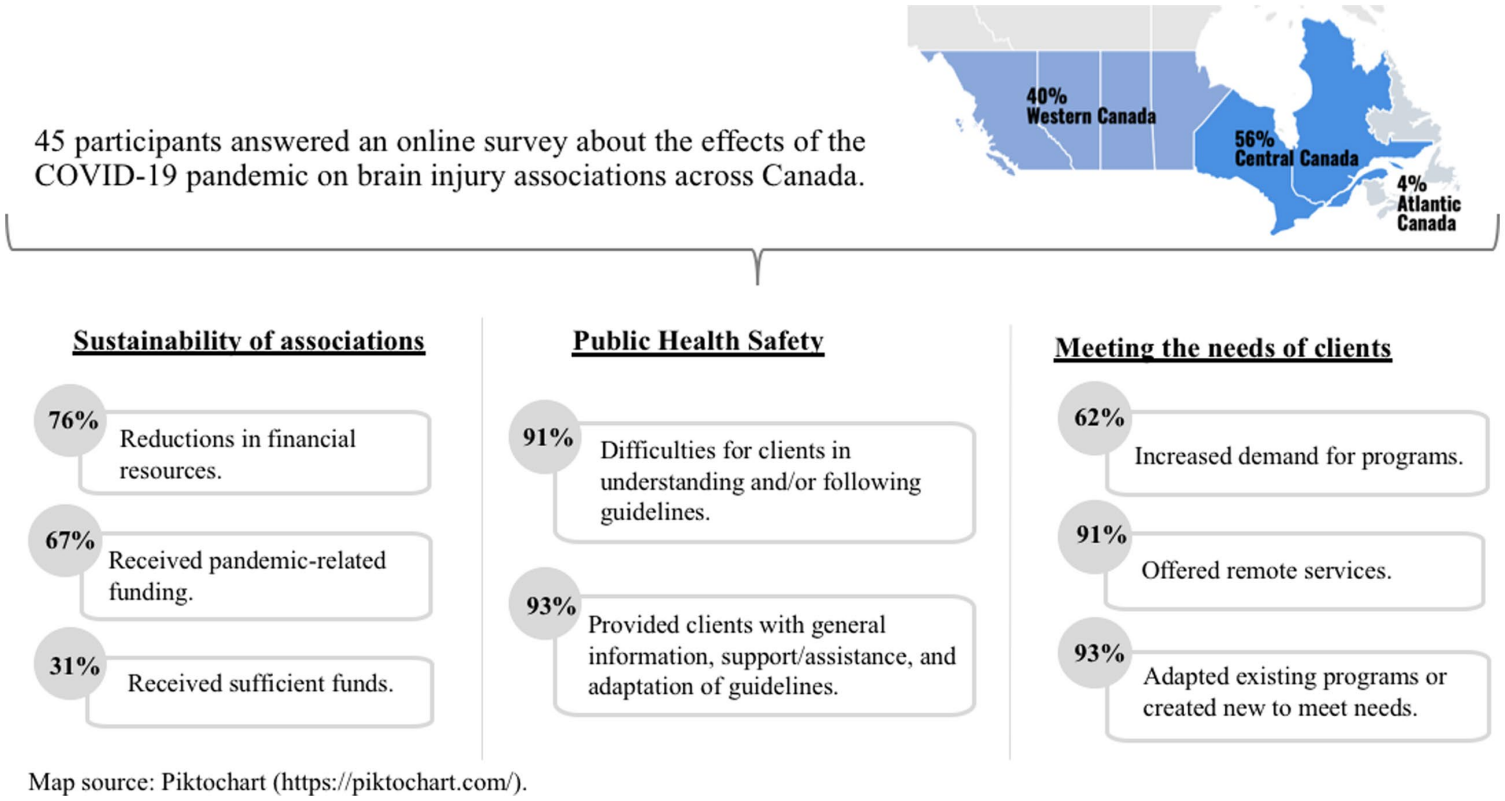
Summary of the main findings of a survey answered by staff or volunteers representatives of brain injury associations across Canada.

Our analyses focused on three key issues related to brain injury associations during the COVID-19 pandemic: challenges experienced by brain injury survivors, adaptation of services during the pandemic, and sustainability of brain injury associations as vital parts of the brain injury care continuum in Canada. A summary of the main findings is presented in Figure 1.

### 3.1. Challenges experienced by brain injury survivors during the COVID-19 pandemic

#### 3.1.1. Psychosocial challenges

According to the participants, clients of their association living with brain injury experienced diverse challenges during the COVID-19 pandemic including (a) social isolation (44; 98%); (b) activity deprivation (43; 96%); (c) loneliness (43; 96%); (d) boredom (42; 93%); (e) anxiety (42; 93%); (f) depression (40; 89%); (g) insecurity about personal finances (35; 78%), food (30; 67%), and housing (26, 58%); and (h) substance use/addiction (25; 56%).

#### 3.1.2. Online challenges

Most of the responding associations (41; 91%) reported that clients who live with brain injury experienced significant challenges in accessing online programs and services provided by the associations. The online challenges reported per association had a low positive correlation with the number of clients to whom brain injury associations provided services before the pandemic (*r*_s_ = 0.37; *p* = 0.022) (Table 2). This means that those associations serving a larger number of clients reported that their clients experienced more difficulties in accessing online services. No significant correlation was found between the number of staff and the online challenges (*r*_s_ = 0.02; *p* = 0.90).

**Table 2:** Correlations between the size of the associations (number of paid staff and number of clients) and (A) meeting the needs of clients, (B) public health safety, and (C) sustainability of brain injury associations.

(A) Meeting the needs of clients								
	Increased demand for services		Offering remote services		Clients experiencing online challenges		Associations ability to address clients’ online challenges	
	Cor.Spearman	*p*-value	Cor.Spearman	*p*-value	Cor.Spearman	*p*-value	Cor.Spearman	*p*-value
Number of staff in the associations	−0.01	0.97	0.14	0.40	0.02	0.90	0.33	0.04*
Number of clients served by the associations (pre-COVID-19)	0.04	0.80	−0.12	0.45	0.40	0.02*	0.05	0.78

(B) Public health safety				
	Clients experiencing challenges with public health guidelines		Associations ability to address public health guidelines challenges experienced by clients	
	Cor.Spearman	*p*-value	Cor.Spearman	*p*-value
Number of staff in the associations	−0.09	0.58	0.16	0.32
Number of clients served by the associations (pre-COVID-19)	−0.08	0.64	0.02	0.92

(C) Sustainability of brain injury associations				
	Loss of funding during the pandemic		Sufficient funding received during the pandemic	
	Cor.Spearman	*p*-value	Cor.Spearman	*p*-value
Number of staff in the associations	−0.19	0.25	0.04	0.83
Number of clients served by the associations (pre-COVID-19)	−0.33	0.04*	0.04	0.79

*p < 0.05.

#### 3.1.3. Challenges following public health guidelines

As public health guidelines changed during different waves of COVID-19, thirty (67%) participants reported that clients experienced challenges in understanding and/or following public health guidelines during the pandemic (e.g., guidelines about social distancing, mask wearing, or hand washing). Correlations with number of staff or clients regarding challenges to understand and follow public health guidelines were not significantly correlated (number of staff: *r*_s_ = −0.09; *p* = 0.58; number of clients: *r*_s_ = −0.08; *p* = 0.64) (Table 2).

### 3.2. Adaptations made by brain injury associations

Most of the associations (42; 93%) quickly adapted pre-existing programs or created new ones, with almost two-thirds of the associations (28; 62%) reporting an increase in demand for support. The increase in demand for services was not significantly correlated with the number of clients (*r*_s_ = 0.04; *p* = 0.80) nor the number of staff (*r*_s_ = −0.01; *p* = 0.97).

One of the biggest changes observed in service delivery was related to the use of remote programs, such as teleservices or online programming. Of responding associations, 91% (*n* = 42) provided remote services with the majority (33; 79%) beginning these services only after the onset of the pandemic. Regarding telephone services, 98% (*n* = 44) of the associations offered these services during the pandemic. However, the use of teleservices was not new to many associations, as 59% (*n* = 26) were already offering this prior to the COVID-19 pandemic, with many associations increasing their use during the current public health crisis. We did not find significant correlations between the offer of remote services and the number of clients (*r*_s_ = −0.12; *p* = 0.45) nor the number of staff (*r*_s_ = 0.14; *p* = 0.40).

The specific adaptations or responses associations made to meet client needs are highlighted in more depth in the open-ended question content analysis (Tables 3 4).

**Table 3:** Quantitative and qualitative content results.

Survey question	Quantitative results (*n*; %)		Qualitative content category	Quotes
For each item, please indicate if your clients experienced a challenge with any of the following:	Social isolation	(Yes – 44; 97.78%)	Reducing psychosocial challenges	Open-ended question: If answering option “yes” to any of the challenge areas, what did your organization do to help alleviate [psychosocial] challenges for your clients during the pandemic? Response: *“We did phone and door check-ins, provided more counseling sessions, liaison with government agencies, community agencies. Added services such as mindfulness and yoga by ZOOM and encouraged participation in ZOOM support groups, provided food and lunch by take out, and delivery. Did check-ins while providing healthy foods. Spoke to landlords [...]. Provided propane and housing comforts to the homeless. Provided grocery gift cards to clients in need” (P02)*
	Loneliness	(Yes - 43; 95.56%)		
	Boredom	(Yes – 43; 95.56%)		
	Activity deprivation	(Yes – 42; 93.33%)		
	Anxiety	(Yes – 42; 93.33%)		
	Depression	(Yes – 40; 88.89%)		
	Financial insecurity	(Yes – 35; 77.78%)		
	Food insecurity	(Yes – 30; 66.67%)		
	Housing insecurity	(Yes – 26; 57.78%)		
	Substance use	(Yes – 25; 55.56%)		
Was your association able to address your clients’ challenges with access to online services?	To a very large extent	(7; 15.56%)	Reducing technological challenges	Open-ended question: Please provide some examples of what you did to meet your clients’ challenges. Response: “*Many clients do not use social media or online services and were/still aren’t comfortable using video conferencing. Others were open to learning so we did spend time teaching things like online banking, ZOOM,* etc. *We also provided Chromebooks and smartphones to those that did not have any access to technology and helped several of them apply for cheaper internet/phone packages through provincial providers…. A phone “tree” system worked to keep people connected without overwhelming staff. Creative solutions were developed to engage non-online participants including*….” (P12).
	To a large extent	(9; 20%)		
	Somewhat	(14; 31.11%)		
	To a small extent	(11; 24.44%)		
Did your association provide services or information to clients explaining public health guidelines?	To a very large extent	(8; 17.78%)	Helping clients to understand and follow protective measures	Open-ended question: What services were provided? Response: “*Informational sheets emailed to participants involved in in-person peer group meetings, activities,* etc. *In-person check for double vaccination verification to enter buildings, requirement masks to be worn at all times, temperature check for anyone entering premises, questionnaire - series of mandatory questions to be answered as part of screening process to enter building….”* (P24).
	To a large extent	(14; 31.11%)		
	Somewhat	(9; 20%)		
	To a small extent	(11; 24.44%)		

**Table 4:** Categories and subcategories related to the community brain injury associations’ strategies to adapting the provision of services to meet the needs of brain injury survivors, their families, and caregivers during the COVID-19 pandemic.

Category of content	Subcategory	Occurrences	Examples
Reducing psychosocial challenges	Psychosocial support	29	Peer group or individual support meetings (social work support, psychologist, home visits) Counselling, referrals to shrinks and medical Online support with link to resources Liaison with government agencies, community agencies and advocacy
	Online activities/ programming	21	Increasing online activities New online programming/activities: Special events, conferences, and workshops Social media groups
	Wellness checks	19	Phone calls, text, or visits to check-in on clients to know how they were feeling and their needs
	Basic needs support	12	Helping with food insecurity (provision of food, food banks, grocery shopping for clients - partnership for essentials supplies) Helping with finance insecurity (banking, gift cards) Helping with housing insecurity (propane and housing for homeless) Wellness kits with masks, hand sanitizer
Reducing technological challenges	Support and/or assistance	29	Workshops and trainings on how to use technology in general (explanatory videos and material with less visual stimulation and provision of step-by-step manuals) Troubleshooting by phone or in-person (clients could use onsite technology) Assistance to apply to fundings and/or cheaper internet and phone packages Helping clients to access programs and/or services external to the association (connection of members with other services providers and resources they were unable to access)
	Provision of internet and/or devices	14	Donations of computers, tablets, chrome books, smartphones Help to cover phone and internet costs
	Other forms of communication (besides online)	8	Option to call in Service delivery in person, doorstep visits, by email, phone, outdoor activities
Helping clients to understand and follow protective measures	General information about COVID-19	35	Education about COVID-19 and restrictions in place Updates regarding changes in guidelines Link to resources General information by phone, email, social media, mail, newsletter, printed material, online meetings
	Assistance and/or support	17	Public health guidance (explanation of how to use masks and the health guidelines) Reminders about rules in place Outreach by phone (to make sure people were following public health measures) Assistance with vaccination (appointments, transport, accompaniment, support with accessing and using QR codes) Provision of masks and hand sanitizer
	Adaptation of public health guidelines	5	Simplification of public health messages Aphasia-friendly COVID-19 screening Modification of written materials Development of new protocols for in person meetings, activities

#### 3.2.1. Reducing psychosocial challenges

To alleviate the extra psychological challenges experienced by people living with brain injury, such as social isolation, loneliness, boredom, anxiety, depression, and insecurities related to finance, housing, and food, associations changed their activities/programming, implemented wellness checks, and provided psychosocial and material support as shown in Tables 3 4.

#### 3.2.2. Reducing online challenges

Forty-one (91%) of the respondents reported that their associations were able to address clients’ challenges related to online services. Brain injury associations attempted to address the challenges experienced by their clients related to access and use of technology by providing clients with devices, support and assistance to access online programs, helping them apply for cheaper internet and phone packages, and creating other forms of communication besides online (Table 4).

The ability to respond to online challenges had a low positive correlation with the number of paid staff (*r*_s_ = 0.33; *p* = 0.040) (Table 2) meaning that those associations that had more paid staff were more able to address their clients’ challenges with accessing online services. The number of clients did not correlate with the ability of the associations to address online challenges (*r*_s_ = 0.05; *p* = 0.78).

#### 3.2.3. Helping clients to understand and follow protective public health guidelines

Many associations reported ongoing efforts to assist their clients with public health guidelines by discussing with them social distancing, sanitizing, and the importance of face masks (Table 4). They also provided clients with information about COVID symptoms and transmission, and ensured that all COVID-19 public safety protocols as dictated by local, provincial, and national public health offices/agencies were followed when meeting in person.

The ability of associations to address public health guideline challenges presented by clients was not correlated with the number of clients (*r*_s_ = 0.02; *p* = 0.92) nor with the number of staff (*r*_s_ = 0.16; *p* = 0.32).

### 3.3. Sustainability of brain injury associations

During the COVID-19 pandemic, the Canadian federal government and many provinces provided additional funding to not-for-profit associations to support their sustainability. Most of the associations completing the survey (30; 67%) applied for and received additional financial support. However, only slightly more than half (25; 56%) reported having received sufficient funding to address additional COVID-19-related expenses. Having received sufficient funding was not related to the number of clients (*r*_s_ = 0.04; *p* = 0.79) nor to the number of staff (*r*_s_ = 0.04; *p* = 0.83).

Despite COVID-19 financial support, participants reported struggling to sustain their associations while adapting their programs and services to meet the needs of their clients. With the widespread sudden cancelation of fundraising events and decreases in financial support from their standard sources, 34 of the responding (76%) associations had significant reductions in funding or financial resources that prevented them from delivering programs or required them to use reserve funds to continue to do so. As shown in Table 2, losing funding had a low negative correlation with indicators of association size (number of clients) indicating that the losses in funding were most difficult for smaller associations (*r*_s_ = −0.33; *p* = 0.037). On the other hand, the number of staff did not correlate with the funding loss during the pandemic (*r*_s_ = −0.19; *p* = 0.25).

## 4. Discussion

This is the first survey study to provide insights into how community brain injury associations across Canada responded to the COVID-19 pandemic. The findings highlight the increased challenges experienced by people living with brain injury during the COVID-19 pandemic. Further, our results show the challenges and threats associations faced to meet clients’ needs and stay viable when many other health services providers had to shut down or reduce their provision of services during the COVID-19 pandemic. This knowledge is needed to develop and advocate for resources that could help brain injury associations and their clients during public health crises not only in Canada but also around the world. This information is needed as Canada and the world now critically examine their pandemic response and do the much needed work to address preparedness for future pandemics or public health crises for all citizens, particularly for vulnerable populations (23).

Similar to recent studies, we found that social isolation, loneliness, boredom, and anxiety were reported by the associations as the most frequent psychosocial issues experienced by brain injury survivors during the COVID-19 pandemic (5, 16). Limited access to treatment or social support during the pandemic also contributed to increased mental health issues in this population (24). Within the brain injury population, there are subgroups that face additional disparities, such as women and LGBTQI2SA+ individuals. These subgroups need particular attention as they can be more affected by intimate partner violence, social and financial disparities, and technological accessibility (25). In Canada, while some health providers shut down or limited their provision of services during the pandemic, brain injury associations quickly pivoted their activities/programming to online and created new services to alleviate the extra psychosocial challenges felt by people living with brain injury, their families, and caregivers. Even though participating associations reported having created new programs to tailor them to specific groups of people living with brain injury during the pandemic, just a few offered specific programs for the LGBTQI2SA+ community. More studies are needed to understand the effects of the pandemic and the needs of this and other subgroups of individuals living with brain injury.

However, the provision of remote services generated novel challenges for brain injury survivors, including discomforts with virtual communication in general, limited or lack of technological knowledge, sparse or no Internet connection, and lack of access to technological devices and or support for using them, all of which were already reported as some of the main reasons for this population to not engage in online activities (14). To overcome this digital divide affecting people with disabilities, brain injury associations across Canada proactively provided their clients with devices and helped them apply for accessible internet/phone packages. Our findings highlight the importance of addressing this continued digital divide to offset these challenges and barriers that meant limited program and service access for people living with brain injury during the COVID-19 pandemic. One possible solution could be the development of technology training and new strategies to facilitate the use of the Internet and devices by people with disabilities (26) which in turn could help them cope with the social isolation, fear, and anxiety intensified by the current pandemic (27).

The digital divide has also made public health recommendations less accessible for people living with disabilities (5, 28). Although the World Health Organization has touted online technologies and remote services as a primary way to address the consequences of public health strategies during the COVID-19 pandemic (6), our results emphasize the need to ensure continuity of in-person services for those people with higher needs or those who are unable to communicate online, while respecting protective measures, such as individualized support by phone, porch visits, and doorstep deliveries.

Moreover, even for clients who had online access to public health information, our results highlight significant challenges in understanding and or following the guidelines. This is not surprising as disaster preparedness materials have often been reported as having large amounts of information, with complicated or inaccessible content for people with disabilities (29). Our findings show that brain injury associations had to seek and translate COVID-19-related evidence to provide their clients with reliable and easily understandable information, assistance, support, and adapted written materials to help them follow the public guidelines in place. The dissemination of public health messages in plain language and accessible formats during the COVID-19 pandemic, a standard recommended by experts in the area of public health messaging (28), was not the reality in many provinces of Canada. By working closely with brain injury and other disability associations, particularly those that help individuals with cognitive and learning disabilities, governmental authorities could provide more accessible and understandable public health messages to a diverse population.

In addition, the COVID-19 pandemic, by affecting multiple sources of revenue, has led many community associations to struggle even more to sustain themselves financially, while complying with restrictive measures, maintaining staff’s mental health and well-being, and continuing to meet the needs of thousands of clients, in a manner similar to other non-profits community associations, and social service providers (15, 30–33). The Canadian government released COVID-19-specific funding and most of the surveyed brain injury associations applied for and received financial support to cope with additional costs associated with the pandemic. However, most associations in our study also lost funding or financial resources since the beginning of the pandemic, especially the smaller ones as our results showed they were more likely to lose funding than larger associations during the COVID-19 pandemic.

Furthermore, Kim and Mason suggested that financial reserves are needed to help non-profits absorb the initial impact of a crisis such as the COVID-19 pandemic, reinforcing the importance of a good strategic plan (15). Developing such a plan however requires more human resources and more long-term funding options, which is not the reality of many associations across Canada. Indeed, key representatives in our study reported that pandemic aside, they are continually seeking financial support and organizing activities to raise funds for the sustainability of their associations. In other words, even though they are on the frontline of community care and support for people living with brain injury, their families, and caregivers, these associations generally lack sufficient funding for association sustainability.

Finally, several studies recommend that preparedness responses must be inclusive and accessible for people living with disabilities (11, 12, 28). According to Villeneuve and colleagues community, health, and disability support workers are the people that individuals with a disability would rely on during an emergency. They observed a lack of preparedness and uncertainty among community, health, and disability stakeholders about how to provide this vulnerable population with support during and after an emergency, especially if they were also affected by the same event (34). Similarly, Jesus and colleagues proposed a model for disability-inclusive pandemic responses for stakeholders to prepare ahead. They suggest that people living with a disability, their caregivers/support persons, and advocates must be involved in emergency preparedness, to ensure that it is truly disability inclusive. Secondly, they propose the use of evidence (quantitative or qualitative) on disability disparities to inform and plan (35).

Going forward, key stakeholders (e.g., policymakers, public health authorities, and civil society) should take appropriate action to assist in disaster preparedness and alleviate the barriers experienced by people with disabilities during and after the COVID-19 pandemic (6). Brain injury associations should also be recognized as essential within the brain injury care continuum. Helping community associations in general to achieve more financial security, but especially the small ones, should be seen as crucial to addressing the public health efforts that people living with brain injury or other disabilities need. In addition, having provincial and national brain injury strategies may make a big difference when it comes to financially supporting these associations. Another solution is to create knowledge-sharing spaces and collaborative connections between all brain injury associations across Canada to advocate for better services for people living with chronic brain injury.

The current study contributes to a better understanding of the impact of the COVID-19 pandemic on community-based brain injury associations across Canada and shows that their actions in response to the pandemic were well aligned with the recommendations of the World Health Organization. However, it has some limitations that need to be highlighted. First, even though we had a wide representation of brain injury associations across Canada, the results of the inferential statistics are underpowered and must be used with caution as they are exploratory results. Studies with larger sample sizes will allow for formal hypotheses testing to identify significant relationships and to test the effect of some potential confounding factors which may increase the generalizability of the results. Second, the challenges experienced by brain injury survivors, their families, and caregivers reported here are from the perception of key representatives of brain injury associations. Third, since the survey was anonymous, we were not able to probe for additional information or clarification. Finally, although we obtained rich data on the adaptations in the provision of services made by brain injury associations during the pandemic, the cross-sectional nature of this survey did not allow us to capture whether changes and innovations in services resulting from this health crisis will be maintained over time. However, qualitative studies analyzing the longitudinal effects of the COVID-19 pandemic on community-based associations across Canada are currently underway (18). In addition, an online resource tool providing a comprehensive plan to address subsequent public health crises more effectively is currently under co-development^3^. This resource is expected to improve the resilience and pandemic preparedness of brain injury associations, strengthen their networking, and provide helpful guidelines for similar health conditions or disabilities nationally and internationally.

## 5. Conclusion

This community-partnership research provides a greater understanding of the impact of the COVID-19 pandemic and related public health policies on individuals living with brain injury and the provision of community-based services to this vulnerable population. Even while facing significant threats to their sustainability, community-based brain injury associations across Canada quickly adapted their services/programs to reduce psychosocial and technological challenges, as well as to help thousands of clients to understand and follow public health measures during the pandemic. Similar experiences are likely in other countries and related to other types of disabilities. People living with disabilities, including brain injury, are a large proportion of the population worldwide. However, the lack of recognition, support, and specific planning for community-based brain injury associations that exist to provide care and assistance to individuals living with a brain injury needs to be considered. Collaborative work among brain injury associations across Canada and future research projects involving stakeholders are needed to better support individuals living with brain injury, their families, and caregivers, and to recognize the vital public health services provided by community-based associations across Canada and around the world.

## Data availability statement

The raw data supporting the conclusions of this article will be made available by the authors, without undue reservation.

## Ethics statement

The studies involving humans were approved by Centre for Interdisciplinary Research in Rehabilitation of Greater Montreal (2022–1424) Ethics committee. The studies were conducted in accordance with the local legislation and institutional requirements. The participants provided their written informed consent to participate in this study.

## Author contributions

AS, CB, SL, MM, MG, BS, JS, CL, AB, and LE were all involved in the study design. AS, CB, SL, MM, MG, BS, JS, and LE developed the survey. AS administered the survey, obtained results, and performed statistical analyses. AS, SL, MM, MG, BS, JS, CL, AB, and LE critically discussed the results. AS and SL wrote the initial draft of the manuscript. CB and LE provided feedback on the first draft of the manuscript. All authors made edits and contributions to the final draft and approved the final version of this manuscript.

## Footnotes

^1^https://braininjurycanada.ca/en/brain-injury-associations

^2^https://www.project-redcap.org

^3^https://cbian.ca/
