# Supplementary material for: Impact of the COVID-19 pandemic on community-based brain injury associations across Canada: a cross-sectional survey study
Source: Front Public Health. 2023 Nov 8;11:1166106. doi: 10.3389/fpubh.2023.1166106 (PMC10663341; doi:10.3389/fpubh.2023.1166106)
Supplement: Supplementary file 1 [file Data_Sheet_1.docx]

Supplementary Material

**Impact of the COVID-19 pandemic on brain injury community associations across Canada: A cross-sectional survey study**

**Ana Paula Salazar, Carolina Bottari, Sophie Lecours, Michelle McDonald, Monique A.M. Gignac, Bonnie Swaine, Julia Schmidt, Carolyn Lemsky, Ashley Brosda, Lisa Engel***

*** Correspondence:** Lisa Engel: [lisa.engel@umanitoba.ca](mailto:lisa.engel@umanitoba.ca)

English and French version of the online survey.

| **BIPP – Online Survey Questions (English version)** | | |
| --- | --- | --- |
| 1. **In this section we would like to learn a bit about your organization and your role in it (9 questions).** | | |
| 1. | Where is your brain injury association located?  (*Choose one option)* | - Northern territories (YT, NT, NU) - Western Canada (BC, AB, SK, MB) - Central Canada (ON, QC) - Atlantic Provinces (NB, NS, PE, NL) |
| 2. | Who is the head of your association, **excluding** the board of directors?  (*Choose one option)* | - A paid executive director - A paid staff, but not an “executive director” - A volunteer or unpaid person - I do not know - I do not want to answer |
| 3. | How long have you been involved with brain injury associations in general?  *(Type your answer)* | _________ (insert number in years)  Or  _________ (insert number in months)   - I do not want to answer (skip) |
| 4. | What is your role in the association?  (*Choose one option)* | - A paid executive director - A paid staff, but not an “executive director” - A volunteer or unpaid person - I do not want to answer |
| 5. | Approximately how many **paid** full-time or part-time **staff** were employed by your association prior to the COVID-19 pandemic?  *(Type your answer)* | _________ (insert number)   - I do not know - I do not want to answer (skip) |
| 6. | Approximately how many people were **volunteering** in your association prior to the COVID-19 pandemic?  *(Type your answer)* | _________ (insert number)   - I do not know - I do not want to answer (skip) |
| 7. | Who are the clients to whom your association provides direct services (i.e., direct services that involve human interaction to individuals or groups and can be completed either by phone, online, or in-person)?  *(Choose all that apply)* | - People with acquired brain injury - Caregivers of people with acquired brain injury - Family members of people with acquired brain injury - Others (Please specify__________) - I do not want to answer |
| 8. | Approximately how many clients did your association provide direct services for **in the last year** before the COVID-19 pandemic?  (*Choose one option)* | - 1-50 - 51-100 - 101-199 - 200-399 - 400-599 - 600-799 - 800 or more - I do not know - I do not want to answer |
| 9. | Approximately how many clients does your association provide direct services to now?  (*Choose one option)* | - 1-50 - 51-100 - 101-199 - 200-399 - 400-599 - 600-799 - 800 or more - I do not know - I do not want to answer |
| **2. In this section we would like to learn a bit about your organization’s funding sources during the COVID-19 pandemic (3 questions).** | | |
| 10. | Did your association lose funding or financial resources because of the COVID-19 pandemic restrictions that  (1) prevented your association from delivering programs or services OR  (2) required your association to use any reserve funds to deliver programs or services?  *(Choose one option)* | - Not at all - To a small extent - Somewhat - To a large extent - To a very large extent - I do not know - I do not want to answer |
| 11. | Did your association receive sufficient funding to address any additional expenses associated with the COVID-19 pandemic?  *(Choose one option)* | - Not at all - To a small extent - Somewhat - To a large extent - To a very large extent - I do not know - I do not want to answer |
| 12. | Did your association apply for COVID-19 specific funding grants?  *(Choose all options that apply)* | - Yes, we applied for federal funding grants and received them. - Yes, we applied for provincial funding grants and received them. - Yes, we applied for local funding grants and received them. - Yes, we applied for federal funding grants, but we did not receive them. - Yes, we applied for provincial funding grants, but we did not receive them. - Yes, we applied for local funding grants, but we did not receive them. - No, we did not apply for any funding grants. - I do not know - I do not want to answer   If you did receive COVID-19 specific funding (answered positive for options 1-3 above), please describe how you used the funding.  _________________ (open text answer) |
| 1. **In this section we would like to learn about the incidence of COVID-19 in your association (4 questions)** | | |
| 13. | Are you aware of the number of your clients who contracted COVID-19?  *(Choose one option)* | - Yes - No - I do not know - I do not want to answer   If yes, approximately how many?  ____ (open text answer) |
| 14. | Did any of your association’s clients require hospitalization because of COVID-19 during the pandemic?  *(Choose one option)* | - Yes - No - I do not know - I do not want to answer   If yes, approximately how many? _____ (open text answer) |
| 15. | Did any of your association’s staff or volunteers contract COVID-19 during the pandemic?  *(Choose one option)* | - Yes - No - I do not know - I do not want to answer   If yes, approximately how many?  ____ (open text answer) |
| 16. | Did any of your association’s staff or volunteers require hospitalization because of COVID-19 during the pandemic?  *(Choose one option)* | - Yes - No - I do not know - I do not want to answer   If yes, approximately how many?  ____ (open text answer) |
| **4. In this section we would like to learn about how your clients respond to public health guidelines during the COVID-19 pandemic (2 questions)** | | |
| 17. | Did your clients report challenges understanding or following public health guidelines such as guidelines about social distancing, mask wearing, or hand washing?  *(Choose one option)* | - Not at all - To a small extent - Somewhat - To a large extent - To a very large extent - I do not know - I do not want to answer |
| 18. | Did your association provide services or information to clients explaining public health guidelines?  *(Choose one option)* | - Not at all - To a small extent - Somewhat - To a large extent - To a very large extent - I do not know - I do not want to answer   If answer is endorsed for options 2-5 above, what services were provided?   - ________________ (open text answer) |
| **5. Thank you for your answers so far. We are about halfway through the questions. In this last section, we would like to learn more about the impact of COVID-19 on your programs and services (13 questions).** | | |
| 19. | During the COVID-19 pandemic, what was the demand for your association’s programs and services? Demand could include number of clients OR intensity of services to meet needs.  (*Choose one option)* | - A lot less than before the pandemic - Somewhat less than before the pandemic - About the same as before the pandemic - Somewhat more than before the pandemic - A lot more than before the pandemic - I do not know - I do not want to answer |
| 20. | Did your organization offer telephone services to clients during the COVID-19 pandemic?  (*Choose one option)* | - Not at all - To a small extent - Somewhat - To a large extent - To a very large extent - I do not know - I do not want to answer   **If Yes (answered 2-5 above; *choose one option*):**   - We offered telephone services before the pandemic and kept the same type/amount of phone services. - We offered telephone services before the pandemic and added additional services. - We offered telephone services before the pandemic but had to decrease our services. - We began offering telephone services during the pandemic. - I do not want to answer.   **If No (answered 1 above; *choose one option*):**   - We did not provide telephone services before or during the pandemic. - We provided telephone services before the pandemic but had to stop them during the pandemic. - I do not want to answer. |
| 21. | Did your association offer its services remotely (i.e., online services) during the COVID-19 pandemic?  *(Choose one option)* | - Not at all - To a small extent - Somewhat - To a large extent - To a very large extent - I do not know - I do not want to answer   **If Yes (answered 2-5 above; *choose one option*):**   - We offered remote services before the pandemic and kept the same type/amount of services. - We offered remote services before the pandemic and added additional services. - We offered remote services before the pandemic but had to decrease our services. - We began offering remote services during the pandemic. - I do not want to answer.   **If No (answers 1 above; *choose one option*):**   - We did not provide remote services before or during the pandemic. - We provided remote services before the pandemic but had to stop them during the pandemic. - I do not want to answer. |
| 22. | Did some of your association’s clients experience challenges accessing online programs or services, either from your association or external to your association?  *(Choose one option)* | - Not at all - To a small extent - Somewhat - To a large extent - To a very large extent - I do not know - I do not want to answer   **If yes (answer is 2-5),** please provide some examples of the types of challenges your association’s clients experienced:  _________ *(open text answer)* |
| 23. | Was your association able to address your clients’ challenges with access to online services?  *(Type your answer)* | - Not at all - To a small extent - Somewhat - To a large extent - To a very large extent - Our association did not address online service access for our clients - I do not know - I do not want to answer   **If yes (answer is 2-5)**, please provide some examples of what you did to meet your clients’ challenges  _________________ (open text answer) |
| 24. | For each item below, please indicate if your clients experienced a challenge with any of the following:   1. Boredom 2. Activity deprivation 3. Loneliness 4. Social isolation 5. Anxiety 6. Depression 7. Substance use/addiction 8. Food insecurity 9. Financial insecurity 10. Housing insecurity/homelessness 11. Other, please specify_____   *(Choose one option per challenge area)* | - Yes - No - I do not know - I do not want to answer   If answering option **“yes” to any of the challenge areas**, what did your organization do to help alleviate these challenges for your clients during the pandemic:  _______ (open text answer) |
| 25. | Do you think the gender identity of your clients (being a woman, man, 2-spirit, non-binary, or other) had an influence on their lives and experiences during the COVID-19 pandemic?  *(Choose one option)* | - Yes - No - I do not know - I do not want to answer |
| 26. | Did your association change or innovate its programs or services in response to the COVID-19 pandemic to meet the needs or challenges of your clients during the pandemic?  *(Type your answer)* | - Yes - No - I do not know - I do not want to answer   If yes, please give some examples of the innovative or changes in services you made:  _______________ (open text answer) |
| 27. | Sometimes different groups of people want programming or services to be tailored to different kinds of needs. In creating programming and services during the COVID-19 pandemic, did your association find that the following groups wanted different programs or services?   1. Clients who identify as men 2. Clients who identify as women 3. LGBTQ2S+ clients 4. Younger clients (18-30 years) 5. Older clients (55+) 6. People living with mild brain injury 7. People living with moderate or severe brain injury 8. Caregivers of people living with brain injuries 9. Families of people living with brain injury 10. Other groups (please explain ___________)   *(Choose one option for each different group listed)* | - Yes - No - I do not know - I do not want to answer |
| 28. | Sometimes different groups of people want programming or services to be tailored to different kinds of needs. In creating programming and services during the COVID-19 pandemic, did your association tailor any programs or services to any of the following groups:   1. Clients who identify as men 2. Clients who identify as women 3. LGBTQ2S+ clients 4. Younger clients (18-30 years) 5. Older clients (55+) 6. People living with mild brain injury 7. People living with moderate or severe brain injury 8. Caregivers of people living with brain injuries 9. Families of people living with brain injury 10. Other groups (please explain ___________)   *(Choose one option for each different group listed)* | - Yes - No - I do not know - I do not want to answer |
| 29. | If you were giving advice to other brain injury associations about ways to improve their programming and services during a pandemic, what would it be?  *(Type your answer)* | __________________ (open text answer)   - I do not want to answer |
| - 30. | Is there anything else that you think is important to know about the adaptations your association made during the COVID-19 pandemic to meet the needs of your clients?  *(Type your answer)* | __________________ (open text answer)   - I do not want to answer |
| - 31. | Do you have any comments to add regarding either the challenges experienced, or innovations made by your association in response to the COVID-19 pandemic?  *(Type your answer)* | __________________ (open text answer)   - I do not want to answer |

**BIPP – Sondage en ligne (Version française)**

| 1. **Dans cette section, nous aimerions en apprendre un peu plus sur votre organisation et votre rôle dans celle-ci** **(9 questions).** | | |
| --- | --- | --- |
| 1. | Où se trouve votre association offrant des services aux personnes vivant avec des lésions cérébrales acquises?  *(Choisissez une réponse)* | - Territoires du Nord (YT, NT, NU) - Ouest Canadian (C.-B., Alb., Sask., Man.) - Centre du Canada (ON, QC) - Provinces de l’Atlantique (N.-B., N.-É., ES, T.-N.-L.) |
| 2. | Qui dirige votre association, à **l’exclusion** du conseil d’administration?  *(Choisissez une réponse)* | - Un directeur général rémunéré - Un membre du personnel rémunéré, mais pas un « directeur général » - Un bénévole ou une personne non rémunérée - Je ne sais pas - Je ne veux pas répondre |
| 3. | Depuis combien de temps approximativement êtes-vous impliqué dans une association offrant des services aux personnes vivant avec des lésions cérébrales acquises?  *(Tapez votre réponse)* | _________ (veuillez indiquer le nombre d’années)  ou  _________ (veuillez indiquer le nombre de mois)   - Je ne veux pas répondre (sauter) |
| 4. | Quel est votre rôle au sein de votre association?  *(Choisissez une réponse)* | - Un directeur général rémunéré - Un membre du personnel rémunéré, mais pas un « directeur général » - Un bénévole ou une personne non rémunérée - Je ne veux pas répondre |
| 5. | Environ combien de **personnes** **rémunérées** à temps plein ou à temps partiel travaillaient pour votre association avant la pandémie de COVID-19?  *(Tapez votre réponse)* | _________ (veuillez indiquer le nombre de personnes)   - Je ne sais pas - Je ne veux pas répondre (sauter) |
| 6. | Environ combien de personnes œuvraient comme **bénévoles** dans votre association avant la pandémie de COVID-19?  *(Tapez votre réponse)* | _________ (veuillez indiquer le nombre de personnes)   - Je ne sais pas - Je ne veux pas répondre (sauter) |
| 7. | Qui sont les personnes auxquelles votre association fournit des services directs (c.-à-d. des services qui impliquent une interaction humaine entre deux personnes et qui peuvent être offerts par téléphone, en ligne ou en personne)?  *(Choisissez tout ce qui s’applique)* | - Personnes vivant avec des lésions cérébrales acquises - Proches aidants de personnes vivant avec des lésions cérébrales acquises - Membres de la famille de personnes vivant avec des lésions cérébrales acquises - Autres (Veuillez spécifier __________) - Je ne veux pas répondre |
| 8. | À combien de personnes votre association fournissait-elle des services directs avant la pandémie de COVID-19?  *(Choisissez une réponse)* | - 1-50 - 51-100 - 101-199 - 200-399 - 400-599 - 600-799 - 800 ou plus - Je ne sais pas - Je ne veux pas répondre |
| 9. | À combien de personnes votre association fournit-elle actuellement des services directs?  *(Choisissez une réponse)* | - 1-50 - 51-100 - 101-199 - 200-399 - 400-599 - 600-799 - 800 ou plus - Je ne sais pas - Je ne veux pas répondre |
| **2. Dans cette section, nous aimerions en apprendre un peu plus sur les sources de financement de votre organisation pendant la pandémie de COVID-19 (3 questions).** | | |
| 10. | Votre association a-t-elle perdu du financement ou des ressources financières en raison des restrictions imposées par la pandémie de COVID-19, soit : (1) en l’empêchant d’offrir des programmes ou des services OU (2) en l’obligeant à utiliser ses fonds de réserve pour offrir des programmes ou des services?  *(Choisissez une réponse)* | - Pas du tout - Dans une faible mesure - Un peu - Dans une large mesure - Dans une très large mesure - Je ne sais pas - Je ne veux pas répondre |
| 11. | Votre association a-t-elle reçu des fonds suffisants pour faire face aux dépenses supplémentaires associées à la pandémie de COVID-19?  *(Choisissez une réponse)* | - Pas du tout - Dans une faible mesure - Un peu - Dans une large mesure - Dans une très large mesure - Je ne sais pas - Je ne veux pas répondre |
| 12. | Votre association a-t-elle fait une ou des demande(s) de subvention spécifiquement liée(s) à la pandémie de COVID-19?  *(Choisissez tout ce qui s’applique)* | - Oui, nous avons présenté une demande de subvention fédérale et nous l’avons obtenue. - Oui, nous avons présenté une demande de subvention provinciale et nous l’avons obtenue. - Oui, nous avons présenté une demande de financement local et nous l’avons obtenu. - Oui, nous avons présenté une demande de subvention fédérale, mais nous ne l’avons pas obtenue. - Oui, nous avons présenté une demande de subvention provinciale, mais nous ne l'avons pas obtenue. - Oui, nous avons présenté une demande de financement local, mais nous ne l'avons pas obtenu. - Non, nous n’avons fait aucune demande de subvention - Je ne sais pas - Je ne veux pas répondre   Si vous avez reçu un financement spécifiquement lié à la pandémie de COVID-19 (une ou plusieurs réponse(s) cochée(s) parmi les options 1 à 3 ci-dessus), veuillez décrire comment vous l’avez utilisé: ________________________________  (réponse en texte ouvert) |
| **3. Dans cette section, nous aimerions en apprendre davantage sur l’incidence de la COVID-19 parmi les membres de votre association (4 questions)** | | |
| 13. | Savez-vous combien de personnes desservies par votre association ont contracté la COVID-19?  *(Choisissez une réponse)* | - Oui - Non - Je ne sais pas - Je ne veux pas répondre   Si oui, combien de clients environ? ____  (réponse en texte ouvert) |
| 14. | Est-ce que des personnes desservies par votre association ont dû être hospitalisées en raison de la COVID-19 pendant la pandémie?  *(Choisissez une réponse)* | - Oui - Non - Je ne sais pas - Je ne veux pas répondre   Si oui, combien de clients environ? ____  (réponse en texte ouvert) |
| 15. | Est-ce que des membres du personnel ou des bénévoles de votre association ont contracté la COVID-19 pendant la pandémie?  *(Choisissez une réponse)* | - Oui - Non - Je ne sais pas - Je ne veux pas répondre   Si oui, combien de personnes environ? ____  (réponse en texte ouvert) |
| 16. | Est-ce que des membres du personnel ou des bénévoles de votre association ont dû être hospitalisés en raison de la COVID-19 pendant la pandémie?  *(Choisissez une réponse)* | - Oui - Non - Je ne sais pas - Je ne veux pas répondre   Si oui, combien de personnes environ? ____  (réponse en texte ouvert) |
| **4. Dans cette section, nous aimerions en apprendre davantage sur la réaction des personnes desservies par votre association face aux consignes de santé publique pendant la pandémie de COVID-19 (2 questions)** | | |
| 17. | Les personnes desservies par votre association vous ont-elles rapporté avoir eu de la difficulté à comprendre ou à suivre les consignes sanitaires de la santé publique, comme la distanciation sociale, le port du masque ou le lavage des mains?  *(Choisissez une réponse)* | - Pas du tout - Dans une faible mesure - Un peu - Dans une large mesure - Dans une très large mesure - Je ne sais pas - Je ne veux pas répondre |
| 18. | Votre association a-t-elle fourni des services ou de l’information à sa clientèle pour leur expliquer les mesures sanitaires de la santé publique?  *(Choisissez une réponse)* | - Pas du tout - Dans une faible mesure - Un peu - Dans une large mesure - Dans une très large mesure - Je ne sais pas - Je ne veux pas répondre   **Si oui** (choix de réponses 2 à 5 ci-dessus), quels services ont été fournis? ________________________________  (réponse en texte ouvert) |
| **5. Merci pour vos réponses jusqu’à présent. Vous avez complété à peu près la moitié des questions. Dans cette dernière section, nous aimerions en apprendre davantage sur l’impact de la COVID-19 sur vos programmes et services (13 questions).** | | |
| 19. | Pendant la pandémie de COVID-19, quelle était la demande pour les programmes et les services de votre association? Cette demande peut se traduire par le nombre de personnes ayant reçu des services OU par l’intensité des services offerts pour répondre aux besoins.  *(Choisissez une réponse)* | - Beaucoup plus qu’avant la pandémie - Un peu plus qu’avant la pandémie - À peu près la même chose qu’avant la pandémie - Un peu moins qu’avant la pandémie - Beaucoup moins qu’avant la pandémie - Je ne sais pas - Je ne veux pas répondre |
| 20. | Votre organisation a-t-elle offert des services au téléphone à sa clientèle pendant la pandémie de COVID-19?  *(Choisissez une réponse)* | - Pas du tout - Dans une faible mesure - Un peu - Dans une large mesure - Dans une très large mesure - Je ne sais pas - Je ne veux pas répondre   **Si oui** (choix de réponses 2 à 5 ci-dessus), choisissez une des options suivantes:   - Nous offrions déjà des services au téléphone avant la pandémie et nous avons continué à le faire de la même façon et au même rythme. - Nous offrions déjà des services au téléphone avant la pandémie et nous avons ajouté des services supplémentaires. - Nous offrions déjà des services au téléphone avant la pandémie, mais nous avons dû diminuer ces services. - Nous avons commencé à offrir des services au téléphone pendant la pandémie. - Je ne veux pas répondre.   **Si non** (choix de réponse 1 ci-dessus), choisissez une des options suivantes :   - Nous n’avons pas fourni de services au téléphone avant ni pendant la pandémie. - Nous fournissions des services au téléphone avant la pandémie, mais nous avons dû les interrompre pendant la pandémie. - Je ne veux pas répondre. |
| 21. | Votre association a-t-elle offert ses services à distance (c.-à-d. des services en ligne) pendant la pandémie de COVID-19?  *(Choisissez une réponse)* | - Pas du tout - Dans une faible mesure - Un peu - Dans une large mesure - Dans une très large mesure - Je ne sais pas - Je ne veux pas répondre   **Si oui** (choix de réponses 2 à 5 ci-dessus), choisissez une des options suivantes:   - Nous offrions déjà des services à distance avant la pandémie et nous avons continué à le faire de la même façon et au même rythme. - Nous offrions déjà des services à distance avant la pandémie et nous avons ajouté des services supplémentaires. - Nous offrions déjà des services à distance avant la pandémie, mais nous avons dû diminuer ces services. - Nous avons commencé à offrir des services à distance pendant la pandémie. - Je ne veux pas répondre.   **Si non** (choix de réponse 1 ci-dessus), choisissez une des options suivantes:   - Nous n’avons pas fourni de services à distance avant ni pendant la pandémie. - Nous avons fourni des services à distance avant la pandémie, mais nous avons dû les interrompre pendant la pandémie. - Je ne veux pas répondre. |
| 22. | Certaines personnes desservies par votre association ont-elles eu de la difficulté à accéder à des programmes ou à des services en ligne, dispensés soit par votre association ou par une organisation extérieure?  *(Choisissez une réponse)* | - Pas du tout - Dans une faible mesure - Un peu - Dans une large mesure - Dans une très large mesure - Je ne sais pas - Je ne veux pas répondre   **Si oui** (choix de réponses 2 à 5 ci-dessus), veuillez fournir quelques exemples des types de défis que les personnes desservies par votre association ont dû relever:  _________ *(réponse en texte ouvert)* |
| 23. | Votre association a-t-elle été en mesure d’aider les personnes qu’elle dessert à relever les défis d’accès aux services en ligne ?  *(Choisissez une réponse)* | - Pas du tout - Dans une faible mesure - Un peu - Dans une large mesure - Dans une très large mesure - Je ne sais pas - Je ne veux pas répondre   **Si oui** (choix de réponses 2 à 5 ci-dessus), veuillez fournir quelques exemples de ce que vous avez fait pour aider vos clients à relever ces défis:   - _________ (réponse en texte ouvert) |
| 24. | Parmi les situations préoccupantes énumérées ci-dessous, veuillez indiquer toutes celles qui ont constitué un défi pour les personnes à qui votre association offre des services :   1. L’ennui 2. La privation d’activité 3. La solitude 4. L’isolement social 5. L’anxiété 6. La dépression 7. La dépendance et l’usage de substances 8. L’insécurité alimentaire 9. L’insécurité financière 10. L’insécurité à l’égard du logement/itinérance 11. Autre, s’il vous plaît spécifiez___   *(Associez une réponse à chacune des situations énumérées)* | - Oui - Non - Je ne sais pas - Je ne veux pas répondre   Si vous avez **répondu « oui », c’est-à-dire que l’une ou l’autre de ces situations a constitué un défi pour les personnes que vous desservez,** veuillez indiquer ce que votre organisation a fait pour les aider à atténuer ce défi pendant la pandémie:  _______ (réponse en texte ouvert) |
| 25. | Pensez-vous que l’identité de genre de des personnes desservies par votre association (femme, homme, bisexuel, non binaire, ou autre) a exercé une influence sur leurs expériences pendant la pandémie de COVID-19?  *(Choisissez une réponse)* | - Oui - Non - Je ne sais pas - Je ne veux pas répondre |
| 26. | Votre association a-t-elle innové dans ses programmes ou ses services ou les a-t-elle adaptés en vue de répondre aux besoins des personnes qu’elle dessert ou de les aider à surmonter des défis pendant la pandémie de COVID-19?  *(Tapez votre réponse)* | - Oui - Non - Je ne sais pas - Je ne veux pas répondre   **Si oui**, veuillez fournir quelques exemples d’innovations réalisées ou de changements apportés à vos services: _______________  (réponse en texte ouvert) |
| 27. | Parfois, différents sous-groupes de personnes souhaitent une programmation ou des services adaptés à différents types de besoins. En créant des programmes et des services pendant la pandémie de COVID-19, votre association a-t-elle trouvé que les sous-groupes suivants souhaitaient des programmes ou des services différents de ceux déjà offerts?   1. Personnes s’identifiant en tant qu’hommes 2. Personnes s’identifiant en tant que femmes 3. Personnes LGBTQ2S+ 4. Personnes jeunes (18-30 ans) 5. Personnes âgées (55+) 6. Personnes vivant avec des lésions cérébrales légères 7. Personnes vivant avec des lésions cérébrales modérées ou sévères 8. Proches aidants de personnes vivant avec des lésions cérébrales 9. Membres de familles de personnes vivant avec des lésions cérébrales 10. Autres groupes (veuillez préciser ________)   *(Choisissez une réponse pour chacun des groupes énumérés)* | - Oui - Non - Je ne sais pas - Je ne veux pas répondre |
| 28. | Parfois, différents sous-groupes de personnes souhaitent une programmation ou des services adaptés à différents types de besoins. En créant des programmes et des services pendant la pandémie de COVID-19, votre association les a-t-elle adaptés pour l’un ou l’autre des sous-groupes suivants?   1. Personnes s’identifiant en tant qu’hommes 2. Personnes s’identifiant en tant que femmes 3. Personnes LGBTQ2S+ 4. Personnes jeunes (18-30 ans) 5. Personnes âgées (55+) 6. Personnes vivant avec des lésions cérébrales légères 7. Personnes vivant avec des lésions cérébrales modérées ou sévères 8. Proches aidants de personnes vivant avec des lésions cérébrales 9. Membres de familles de personnes vivant avec des lésions cérébrales 10. Autres groupes (veuillez préciser ________)   *(Choisissez une réponse pour chacun des groupes énumérés)* | - Oui - Non - Je ne sais pas - Je ne veux pas répondre |
| 29. | Si vous aviez à donner des conseils à d’autres associations de personnes présentant des lésions cérébrales acquises sur les façons d’améliorer leurs programmes et leurs services pendant une pandémie, quels seraient-ils?  *(Tapez votre réponse)* | ______ (réponse en texte ouvert)   - Je ne veux pas répondre |
| 30. | Y a-t-il autre chose que vous croyez important de nous faire connaître au sujet des adaptations de services réalisées par votre association pour répondre aux besoins des personnes qu’elle dessert pendant la pandémie de COVID-19?  *(Tapez votre réponse)* | ______ (réponse en texte ouvert)  Je ne veux pas répondre |
| - 31. | Avez-vous des commentaires à ajouter au sujet des défis rencontrés par votre association ou des innovations qu’elle a réalisées en réponse à la pandémie de COVID-19?  *(Tapez votre réponse)* | ______ (réponse en texte ouvert)   - Je ne veux pas répondre |
